# Polyethylene Migration from Food Packaging on Cheese Detected by Raman and Infrared (ATR/FT-IR) Spectroscopy

**DOI:** 10.3390/ma14143872

**Published:** 2021-07-12

**Authors:** Klytaimnistra Katsara, George Kenanakis, Zacharias Viskadourakis, Vassilis M. Papadakis

**Affiliations:** 1 Institute of Molecular Biology and Biotechnology, Foundation for Research and Technology-Hellas, N. Plastira 100, GR-70013 Heraklion, Greece; klytaimnistra_katsara@imbb.forth.gr; 2 Institute of Electronic Structure and Laser, Foundation for Research and Technology-Hellas, N. Plastira 100, GR-70013 Heraklion, Greece; gkenanak@iesl.forth.gr (G.K.); zach@iesl.forth.gr (Z.V.)

**Keywords:** food packaging, polyethylene, polymer migration, Raman spectroscopy, FT-IR spectroscopy

## Abstract

For multiple years, food packaging migration has been a major concern in food and health sciences. Plastics, such as polyethylene, are continuously utilized in food packaging for preservation and easy handling purposes during transportation and storage. In this work, three types of cheese, Edam, Kefalotyri and Parmesan, of different hardness were studied under two complementary vibrational spectroscopy methods, ATR-FTIR and Raman spectroscopy, to determine the migration of low-density polyethylene from plastic packaging to the surface of cheese samples. The experimental duration of this study was set to 28 days due to the degradation time of the selected cheese samples, which is clearly visible after 1 month in refrigerated conditions at 4 °C. Raman and ATR-FTIR measurements were performed at a 4–3–4–3 day pattern to obtain comparative results. Initially, consistency/repeatability measurement tests were performed on Day_0_ for each sample of all cheese specimens to understand if there is any overlap between the characteristic Raman and ATR-FTIR peaks of the cheese with the ones from the low-density polyethylene package. We provide evidence that on Day_14_, peaks of low-density polyethylene appeared due to polymeric migration in all three cheese types we tested. In all cheese samples, microbial outgrowth started to develop after Day_21_, as observed visually and under the bright-field microscope, causing peak reverse. Food packaging migration was validated using two different approaches of vibrational spectroscopy (Raman and FT-IR), revealing that cheese needs to be consumed within a short time frame in refrigerated conditions at 4 °C.

## 1. Introduction

Polymeric and plastic packaging has been directly linked to food for decades, as they ensure their delicious, nutritional and aesthetic characteristics while maintaining their nutritional properties for a longer time. A variety of materials have been proposed in the past to preserve and transport food easily, while several research efforts have been focused on innovative technologies and packaging solutions, such as intelligent, recyclable, easy-to-use and antibacterial packing solutions, most of which are polymers, often referred to as plastics [1,2,3]. There are plenty of plastics, but only some of them are utilized in food packaging; the most common are polyethylene (PE), polyamide (nylon), polyethylene terephthalate (PET), polypropylene (PP), polyvinylidene chloride (PVDC), etc. [1,3]. Among the plastics used in food packaging, mentioned above, PE and especially low-density polyethylene (LDPE) are thinner than some other polymers, while they remain stable up to high temperatures. Due to its toughness and flexibility, LDPE is primarily used in film applications where heat sealing is needed, but it is also used in rigid applications and thus can be used for various types of food packaging, such as beverage, soup wrappers, fruit and vegetable bags used in grocery stores, as well as vacuum packaging of cheese and dairy products [2,4]. However, the nature of the influence of the above plastics on food is an open research question, and the suspicion of compound migration remains to be fully addressed. The term “migration” refers to the diffusion of substances from food packaging to the food surface [3,5,6,7,8,9,10,11,12]. In this work, two different vibrational spectroscopic methods (ATR/FT-IR and Raman) were tested to determine the migration of LDPE from plastic packaging to three types of cheese, namely Edam (soft cheese), Kefalotyri (Cretan/Greek semihard cheese) and Parmesan (hard cheese).

We present evidence that that LDPE is indeed migrating from food packaging to all cheese samples, while kept in refrigeration temperatures (~4 °C), and migration is detectable by the spectroscopic techniques from Day 14.

## 2. Materials and Methods

### 2.1. Samples and Experiment Preparation

The experimental design was based on the selected cheese degradation time, which is clearly visible after 1 month in refrigerated conditions at 4 °C. For this reason, the experimental duration was set to 28 days, with consecutive time intervals of 3 and 4 days to allow measurements always within working days. In particular, the measurement days alternated at 4–3–4–3 day pattern (2 measurement days per week, always on Mondays and Thursdays). This approach resulted in 9 time points for every cheese, named Day_0_, Day_4_, Day_7_, Day_11_, Day_14_, Day_18_, Day_21_, Day_25_ and Day_28_.

**Figure 1:**
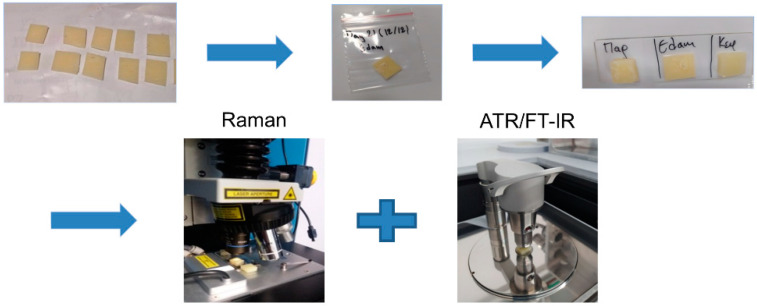
Sample preparation process.

Twenty-four resealable air-tight food-grade LDPE pouches, single-layered of 170 μm thickness, were bought from a local distributor. They were prepared and premarked with the assigned measurement day and cheese type. Three different kinds of cheese, Edam (soft cheese, fat: 29 g/100 g), Kefalotyri (Cretan semihard cheese, fat: 25 g/100 g) and Parmesan (hard cheese, fat: 28 g/100 g), were prepared for this experiment. Initially, all cheese surfaces touching the packaging were removed. The cleaned cheese was cut into small square pieces and then placed into the premarked LDPE pouches as shown in Figure 1. Air was manually removed (by hand pressure) from the cheese–LDPE interface, ensuring direct contact, and then pouches were well sealed. To avoid any possibility of polymer migration at Day_0_, the first sample from each cheese type was measured right after cutting. For reference purposes, the cheese surface spectral information was acquired on Day_0_, where cheese surface was intact, and in particular without having touched the LDPE pouch. The remaining samples were stored inside a refrigerator at 4 °C and measured on the corresponding measurement day premarked on the LDPE pouch.

### 2.2. Data Acquisition

Data acquisition was performed with three state-of-the-art instruments located in our premises. In particular, we used a modified Raman microscope (LabRAM HR; HORIBA FRANCE SAS, Longjumeau, France), an ATR/FT-IR spectrometer (Vertex 70v; Bruker Optik GmbH, Rosenheim, Germany) coupled with a Bruker A225/Q Platinum ATR unit (Bruker Optik GmbH, Rosenheim, Germany) with single reflection diamond crystal and an X-ray diffractometer (D8 Advance Bruker Optik GmbH, Rosenheim, Germany).

#### 2.2.1. Instrument Description and Acquisition Settings

##### LabRAM HR Raman Microscope

A LabRAM HR confocal Raman microscope equipped with an excitation laser line of a central wavelength at 532 nm and a laser output power of ~100 mW. The objective lens used was an Olympus 50× lens with a numerical aperture (NA) of 0.5 and a working distance of 10.6 mm (LMPlanFLN 50X/0.5, Olympus, Tokyo, Japan). The resulting maximum laser power on the sample under the aforementioned setup was measured to be 33 mW with a laser spot of ~1.3 μm. A grating of 600 groves was used that resulted in a Raman spectral resolution of around 2 cm^−1^_._ The Raman signal detector was a Syncerity CCD Deep Cooled Camera by Horiba, operating at −50 °C. A temperature-controlled stage (PE120-XY, Linkam Scientific Instruments Ltd.; Surrey, UK) coupled with the microscope stage, ensured sample’s temperature control and stability. Instrument calibration was performed before each experiment; spectral calibration was performed with a Si reference sample, presenting a single peak at 520.7 cm^−1^. The microscope was set to acquire Raman spectra from 300 up to 3150 cm^−1^, while the 532 nm excitation laser operated at maximum intensity (100%) resulting in 33 mW on the sample. Acquisition time was set to 10 s, and there were 3 spectral accumulations, resulting in a total acquisition time of around 30 s for every point. All measurements were performed under a constant temperature at 18 °C, using a Linkam PE120 thermoelectrically cooled stage. At the time of measurement, samples were placed onto a glass microscope slide using forceps, maintaining the orientation they had in the LDPE pouches. From each sample, more than 3 measurements at different points were acquired to check for Raman signal consistency. All measurements were acquired around the middle area of the cheese samples.

##### ATR/FT-IR

ATR/FT-IR (absorbance) experiments were carried out using a Bruker Vertex 70v FT-IR vacuum spectrometer, equipped with an A225/Q Platinum ATR unit with single reflection diamond crystal which allows the infrared analysis of unevenly shaped solid samples through total reflection measurements, in a spectral range of 7500–350 cm^−1^. A broadband KBr beamsplitter (Bruker Optik GmbH, Rosenheim, Germany) and a room temperature broadband triglycine sulfate (DTGS) detector (Bruker Optik GmbH, Rosenheim, Germany) were used, while interferograms were collected at 4 cm^−1^ resolution (8 scans), apodized with a Blackman–Harris function and Fourier transformed with two levels of zero filling to yield spectra encoded at 2 cm^−1^ intervals. Before scanning the samples, a background diamond crystal was recorded, and each sample spectrum was obtained by automatic subtraction of it. For each measurement, the samples were carefully placed under the ATR press, maintaining the orientation they had in the pouches, while after every measurement the sample area and the tip of the A225/Q ATR unit were cleaned with pure ethanol (Et-OH; Sigma-Aldrich, Munich, Germany).

##### XRD

X-ray diffraction (XRD) measurements were performed using a D8 Advance Bruker Optik GmbH (Rosenheim, Germany) diffractometer with Twin-Twin technology, a copper sealed tube X-ray source producing Cu kα radiation at a wavelength of 1.5406 Å, for 2-theta = 20.00–40.00° and a speed of 0.01°/s.

### 2.3. Data Processing and Analysis

#### 2.3.1. Spectral References

##### Raman Spectral References

**Figure 2:**
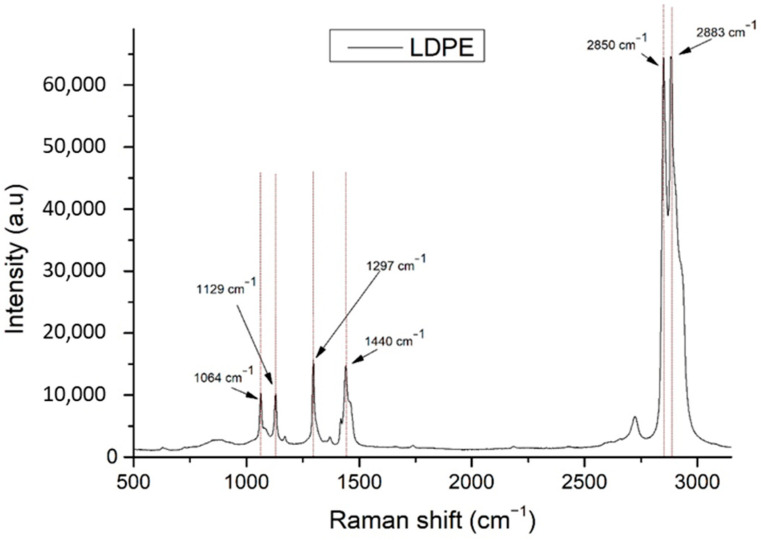
Raman spectrum of LDPE.

The Raman spectrum of LDPE is presented in Figure 2. The major Raman peaks are presented in dashed lines indicating the important Raman peaks on the same graph. The numbering of the Raman peaks is according to ascending wavenumber order.

In Table 1, the identified Raman peaks of LDPE are presented, according to KnowItAll Informatics System by *Bio-Rad Laboratories* database. A literature study was performed to identify the related assignments of those LDPE Raman peaks found in existing cheese studies. These assignments are presented in Table 1, together with the associated references.

##### ATR/FT-IR Spectral References

**Figure 3:**
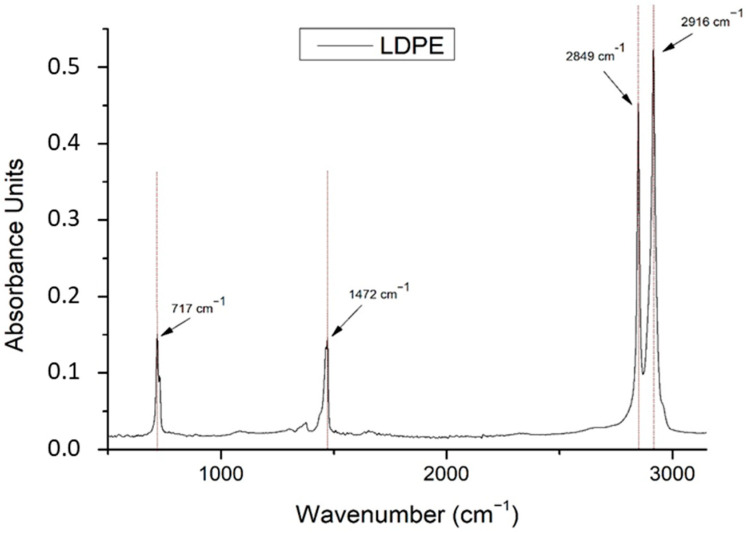
The ATR/FT-IR spectrum of LDPE.

The ATR/FT-IR spectrum of LDPE is presented in Figure 3. The major ATR/FT-IR peaks are presented in dashed lines indicating the important ATR/FT-IR peaks on the same graph. The numbering of the ATR/FT-IR peaks is according to ascending wavenumber order.

In Table 2, the identified ATR/FT-IR peaks of LDPE are presented, as described in the S.T. Japan Europe GmbH FT-IR database. A literature study was performed to identify the related assignments of those ATR/FT-IR peaks found in existing cheese studies; they are also presented together with the associated references.

##### XRD Spectral References

**Figure 4:**
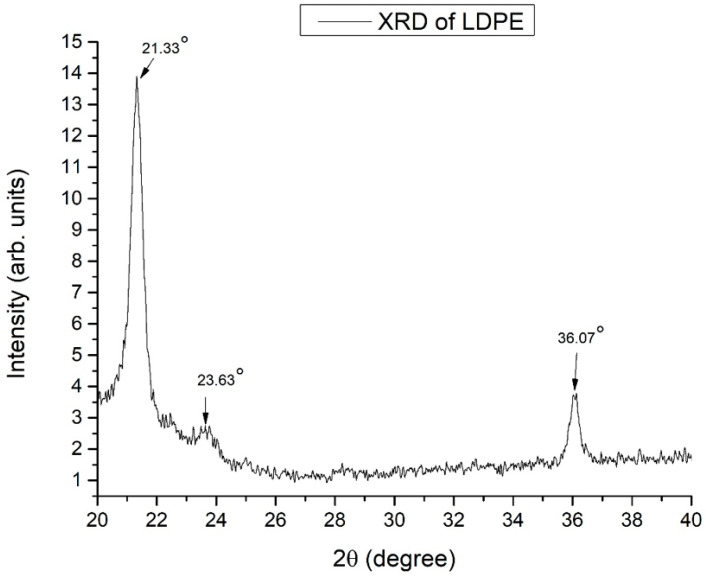
The XRD pattern of LDPE.

The XRD pattern of LDPE is presented in Figure 4. The major XRD peaks are presented with arrows indicating the related diffraction angles.

Figure 4 shows that the XRD peaks are in accordance with the literature [23,24,25,26] and JSPDS card 11-0834, verifying that the packaging material used is indeed LDPE.

In Table 3, the identified XRD peaks of LDPE are presented, as described in [23,24,25,26].

#### 2.3.2. Spectra Processing and Analysis

Raman spectra were acquired, processed, analyzed and visualized by LabSpec 6 Raman software, made by Horiba (HORIBA FRANCE SAS, Longjumeau, France). Additionally, ATR/FT-IR spectra were acquired, processed, analyzed and visualized by Opus 7.2 software, made by Bruker (Bruker Optik GmbH, Rosenheim, Germany). Afterward, all manuscript figures were created with MS Excel (Microsoft Corporation, Washington, DC, USA).

##### Raman Spectra Processing and Analysis

For each Raman spectrum, the following processing methodology was used: (a) cosmic rays were removed, (b) background was removed using a 5th-order polynomial function and (c) final spectrum was smoothed under a Gaussian filter with a kernel of 5 points. Afterward, for each cheese measurement, all Day_x_ Raman spectra were then normalized by the first Day_0_ reference spectrum to observe only the Raman spectral changes throughout the experiment.

##### ATR/FT-IR Spectra Processing and Analysis

For each ATR/FT-IR spectrum, the following processing methodology was used: after performing each cheese measurement, as described in Section 2.3.2, all day (Day_x_) ATR/FT-IR spectra were normalized by the first (Day_0_) reference spectrum to observe only the ATR/FT-IR spectral changes throughout the experiment.

## 3. Results and Discussion

### 3.1. Spectroscopic Analysis (Raman and ATR/FT-IR Spectroscopy)

#### 3.1.1. Consistency/Repeatability Tests

**Figure 5:**
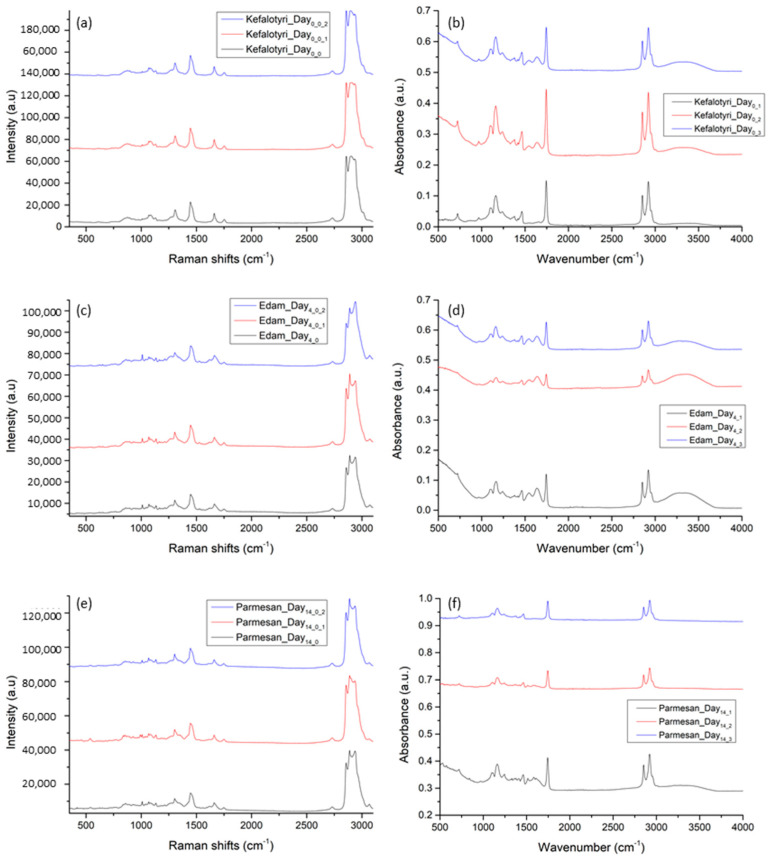
Raman and ATR/FT-IR consistency/repeatability measurements from the 3 different cheese samples: (**a**,**b**) show the Kefalotyri at Day_0_, (**c**,**d**) Edam at Day_4_, and (**e**,**f**) Parmesan at Day_14_. Spectra are presented in stack-line format, with an offset between them, in order to present the full spectral details in each sample. For this reason, the intensity axis is in arbitrary units.

Initially, we performed a consistency/repeatability test of the measurements on the same sample. For this reason, three measurements at different locations were acquired from each cheese sample. These measurements are presented in Figure 5, with an offset between them to show the actual similarities. As it is shown, measurements were very consistent, presenting high repeatability. We followed this test across all the measurements, always acquiring a minimum of three measurements from each sample. In Figure 5, we also present the consistency/repeatability measurements from our three different cheese samples and on three different days (Day_0_, Day_4_ and Day_14_). The standard deviation (SD) of the average at the highest signal peak for each cheese type was at 2889 cm^−1^ for Raman (Kefalotyri 0.44%, Edam 11.58%, Parmesan 7.02%) and at 2921 cm^−1^ for ATR/FT-IR (Kefalotyri 16.7%, Edam 28.15%, Parmesan 50.9).

**Figure 6:**
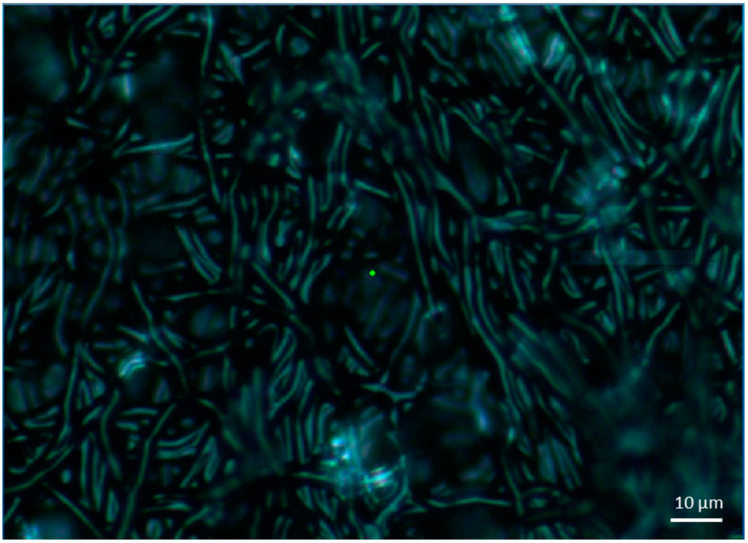
Bright-field picture of the Parmesan cheese sample from Raman microscope on Day_25_.

At this point, we should comment that, some inconsistencies are present in the measurements after Day_21_ that are attributed to the microbial outgrowth (biofilm) that is developed after Day_18_, or cheese surface oxidation. One example of this degradation is presented in Figure 6, which contains a microscopic picture from Parmesan cheese on Day_25_.

#### 3.1.2. LDPE Detection

**Figure 7:**
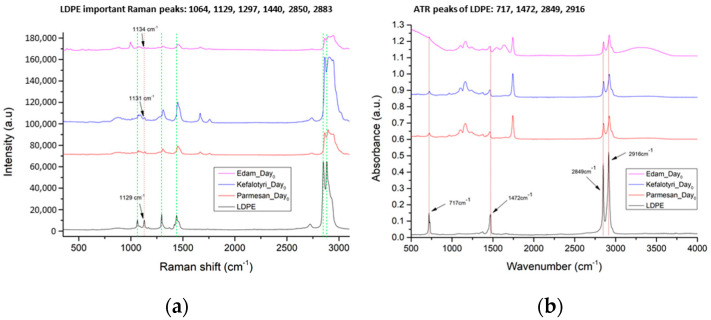
Reference spectra for (**a**) Raman and (**b**) ATR/FT-IR from cheese samples on Day_0_ and LDPE. Spectra are presented in stack-line format, with an offset between them, in order to present the full spectral details in each sample. For this reason, the intensity axis is in arbitrary units.

Initially, we measured all cheese samples on Day_0_ to understand if there is any overlap between the characteristic Raman and ATR/FT-IR peaks of the cheese samples with the ones from the LDPE package. Raman and ATR/FT-IR measurements are presented in Figure 7. As can be seen, the Raman peaks of LDPE are not present in the cheese spectrum, except only one, at 1129 cm^−1^; in Kefalotyri cheese, this peak is present at 1131 cm^−1^, and in Parmesan cheese, it is at 1134 cm^−1^. Although these Raman peaks from the different cheese samples are not presented exactly at the same LDPE peak of 1129 cm^−1^, they are very close to this peak and are considered the same. On the contrary, the ATR/FT-IR peaks of LDPE exist in all cheese samples, so the detection of the LDPE migration was focused on the intensity changes.

**Figure 8:**
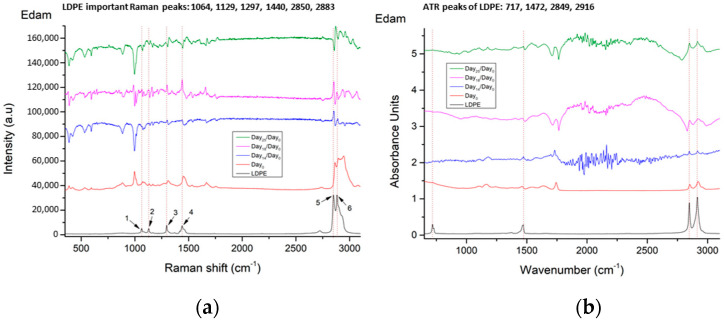
(**a**) Raman spectra and (**b**) ATR/FT-IR spectra, of Edam cheese on different days, in comparison with the LDPE spectrum. Spectra are presented in stack-line format, with an offset between them, in order to present the full spectral details in each sample. For this reason, the intensity axis is in arbitrary units.

In each of the following figures, we present the LDPE spectrum, in black color, together with the Day_0_ Raman and ATR/FT-IR spectra from each cheese type (Edam, Kefalotyri and Parmesan), in red color, as references. Together with the reference spectra, we show the evolution of the Raman and ATR/FT-IR spectral differences from Day_0_, across selected days. In Figure 8, Raman and ATR/FT-IR spectral differences of Day_14_ are presented in blue, those of Day_18_ are presented in purple and those of Day_25_ are presented in green.

As can be seen in the Raman and the ATR/FT-IR spectral differences in Figure 8, from Day_14_, all LDPE peaks start to appear in Edam cheese, and on Day_18_, these peaks become more distinct. The fact that we observe no differences in the first day that LDPE peaks appear in our study restricts us from comparing the differences in fat content between the cheese types as described in related studies [7,27]. On Day_25_, these peaks are reversed, probably due to surface oxidation or the presence of biofilm developing. This is also observed in the cheese visually and under the bright-field microscope, starting from Day_21_.

**Figure 9:**
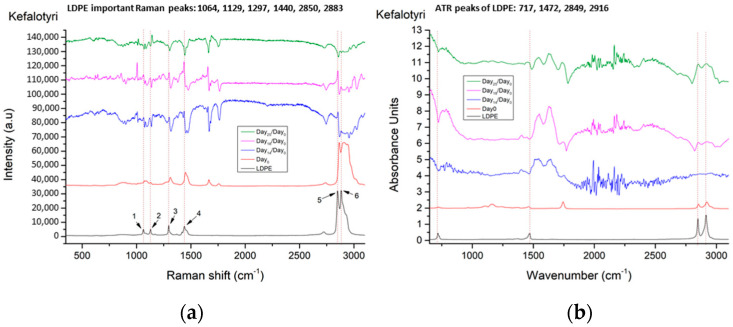
(**a**) Raman and (**b**) ATR/FT-IR spectra, of Kefalotyri cheese on different days, in comparison with the LDPE spectrum. Spectra are presented in stack-line format, with an offset between them, in order to present the full spectral details in each sample. For this reason, the intensity axis is in arbitrary units.

A similar behavior is found in Kefalotyri cheese. As we observe in Figure 9, on Day_14_ and Day_18_ for Raman and ATR/FT-IR spectra, respectively, LDPE peaks appear with a gradual increase across the days. Again, on Day_25_ these peaks are reversed, probably due to oxidation or the presence of the biofilm developing in the cheese surface. This was verified visually and under the bright-field microscope, clearly obvious on Day_25_.

**Figure 10:**
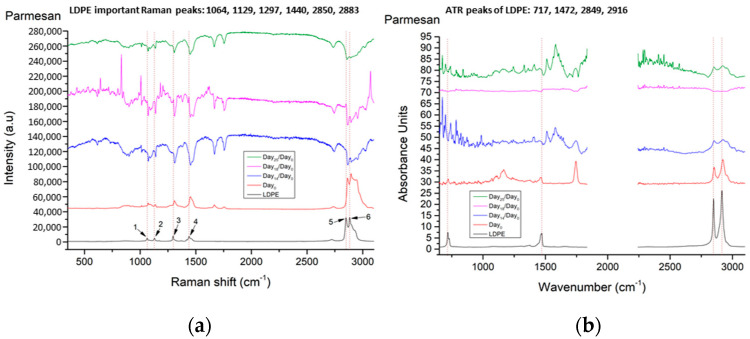
(**a**) Raman and (**b**) ATR/FT-IR spectra, of Parmesan cheese on different days, in comparison with the LDPE spectrum. Spectra are presented in stack-line format, with an offset between them, in order to present the full spectral details in each sample. For this reason, the intensity axis is in arbitrary units.

Parmesan cheese was the most stable one, concerning the oxidation of the cheese surface and the development of the biofilm. As we observe from Figure 10, on Day_14_, LDPE peaks start to appear in both techniques. On Day_18_, these peaks for Raman increase and become more distinct, while no significant changes were observed for the sample used in ATR/FT-IR, probably due to a sample error. On Day_25_, similarly to the other cheese samples, these peaks have been reversed for Raman, although no visible degradation was found visually or under the bright-field microscope. For ATR/FT-IR, we observed an increase in the LDPE peaks. Following the spectral differences from Day_0_, we can be sure that there is no interplay between the LDPE characteristic peaks and the cheese Raman signal behavior throughout the days. Furthermore, the spectral features from both Raman and ATR/FT-IR present in the later experimental days, where oxidation and/or biofilm starts to develop, showing that there is no direct correlation with the LDPE spectral features measured. Taking this into account, together with the fact that all LDPE peaks are present from Day_14_, proves that the Raman and ATR/FT-IR spectra identified are most probably from the polymer itself, with the further understanding that our instruments’ sensitivity managed to detect those peaks only after Day_14_. We make this statement to clarify that LDPE migration might have already been initiated in earlier days, but the concentration of the polymeric migration is not sufficient to be detected with our instrumentation.

## 4. Conclusions

In this work, we show that LDPE migration from food packaging is indeed occurring in three different cheese samples in refrigeration temperatures (4 °C). Based on our experimental setup, we managed to observe this polymeric migration through time, firstly identifying it on Day_14_, in all three cheese types we tested. The LDPE migration was validated with two independent techniques, Raman and ATR/FT-IR spectroscopy. An efficient and simple vibrational spectral measurement methodology is presented that enabled us to achieve fast and robust measurements for LDPE migration detection.

## Figures and Tables

**Table 1:** List of the major Raman peak assignments commonly found in cheese and LDPE.

No.	LDPE Major Raman Peaks (cm^−1^)	Raman Peak Assignments Found in Cheese Studies (cm^−1^)
1	1064	1060 ➔ C-C skeletal stretching vibration out-of-plane [13] 1065 ➔ v (C-C) in fatty acids [14]
2	1129	1125 ➔ C-C skeletal stretching vibration trans chain conformation in plane [13] 1127 ➔ v (C-C) of carotenoids [14] 1132 ➔ rocking of NH_3_^+^ in leucine [15]
3	1297	1296 ➔ CH_2_ twist [13] 1301 ➔ CH_2_ twisting mode of phospholipids (phosphatidylcholine, phosphatidylinositol, phosphatidylserine) [14] 1303 ➔ fat band [14], amide III (protein structures) [16]
4	1440	1440 ➔ deformation modes of the CH_2_ group (in lipids, carbohydrates, amino acids) [17] 1440,1441 ➔ large fat globule, fat band [14] 1442 ➔ CH_2_ scissoring from cholesterol [14] 1444 ➔ lipids [16] 1445 ➔ CH_2_ bend scissoring deformation, amide III [13]
5	2850	2850 ➔ ν_s_ CH_2_, lipids, fatty acids, CH_2_ symmetric (especially for biological tissues) [18] 2852 ➔ vibrational modes of lipids [17]
6	2883	2883 ➔ CH_2_ asymmetric stretch of lipids and proteins (especially for biological tissues) [18] 2880 ➔ vibrational modes of lipids [17]

**Table 2:** List of the major ATR/FT-IR LDPE peaks and their assignments.

No.	LDPE Major ATR/FT-IR Peaks (cm^−1^) [19,20]	ATR/FT-IR Peak Assignments in Cheese Bonds (cm^−1^)
1	717 ➔ rocking vibration of -CH_2_ in LDPE	Does not exist in cheese samples
2	1472 ➔ bending vibrations of -CH_2_ and -CH_3_ in LDPE	950–1490 ➔ carbohydrates (lactose and monosaccharides) [21] 1199–1474 ➔ O-C-H, C-C-H, C-OH vibrational modes from sugars and organic acids [22]
3	2849 ➔ stretching vibration of -CH in LDPE	2850 ➔ C-H stretching of methylene groups [22] 2851 ➔ methylene band (CH_2_ in fat) [21]
4	2916 ➔ stretching vibration of -CH_2_ in LDPE	2920 ➔ C-H stretching of methylene groups [22] 2920 ➔ methylene band (CH_2_ in fat) [21]

**Table 3:** List of the major XRD LDPE peaks and their assignments [23,24,25,26].

No.	LDPE Major Diffraction Angles (°)	LDPE XRD Diffraction Planes (Miller Indices)
1	21.33	(110)
2	23.63	(200)
3	36.07	(020)

## Data Availability

All data reported here can be made available upon request.
